# Lack of evidence for a causal role of *CALR3* in monogenic cardiomyopathy

**DOI:** 10.1038/s41431-018-0208-1

**Published:** 2018-07-09

**Authors:** Judith M. A. Verhagen, Job H. Veldman, Paul A. van der Zwaag, Jan H. von der Thüsen, Erwin Brosens, Imke Christiaans, Dennis Dooijes, Apollonia T. J. M. Helderman-van den Enden, Ronald H. Lekanne Deprez, Michelle Michels, Anneke M. van Mil, Rogier A. Oldenburg, Jasper J. van der Smagt, Arthur van den Wijngaard, Marja W. Wessels, Robert M. W. Hofstra, Marjon A. van Slegtenhorst, Jan D. H. Jongbloed, Ingrid M. B. H. van de Laar

**Affiliations:** 1 Department of Clinical Genetics, Erasmus University Medical Center, Rotterdam, The Netherlands; 2 Department of Genetics, University of Groningen, University Medical Center Groningen, Groningen, The Netherlands; 3 Department of Pathology, Erasmus University Medical Center, Rotterdam, The Netherlands; 4 Department of Clinical Genetics, Academic Medical Center, University of Amsterdam, Amsterdam, The Netherlands; 5 Department of Genetics, University Medical Center Utrecht, Utrecht University, Utrecht, The Netherlands; 6 Department of Clinical Genetics, Maastricht University Medical Center, Maastricht, The Netherlands; 7 Department of Cardiology, Erasmus University Medical Center, Rotterdam, The Netherlands; 8 Department of Clinical Genetics, Leiden University Medical Center, Leiden, The Netherlands

## Abstract

The pathogenicity of previously published disease-associated genes and variants is sometimes questionable. Large-scale, population-based sequencing studies have uncovered numerous false assignments of pathogenicity. Misinterpretation of sequence variants may have serious implications for the patients and families involved, as genetic test results are increasingly being used in medical decision making. In this study, we assessed the role of the calreticulin-3 gene (*CALR3*) in cardiomyopathy. *CALR3* has been included in several cardiomyopathy gene panels worldwide. Its inclusion is based on a single publication describing two missense variants in patients with hypertrophic cardiomyopathy. In our national cardiomyopathy cohort (*n* = 6154), we identified 17 unique, rare heterozygous *CALR3* variants in 48 probands. Overall, our patient cohort contained a significantly higher number of rare *CALR3* variants compared to the ExAC population (*p* = 0.0036). However, after removing a potential Dutch founder variant, no statistically significant difference was found (*p* = 0.89). In nine probands, the *CALR3* variant was accompanied by a disease-causing variant in another, well-known cardiomyopathy gene. In three families, the *CALR3* variant did not segregate with the disease. Furthermore, we could not demonstrate calreticulin-3 protein expression in myocardial tissues at various ages. On the basis of these findings, it seems highly questionable that variants in *CALR3* are a monogenic cause of cardiomyopathy.

## Introduction

Cardiomyopathies are a heterogeneous group of disorders affecting the myocardium. Variants in genes encoding sarcomeric and Z-disc proteins, including cardiac myosin-binding protein C (*MYBPC3*), β-myosin heavy chain (*MYH7*), and titin (*TTN*), account for the majority of cases [1]. Chiu et al. [2] hypothesized that variants in genes encoding calcium-regulating proteins may be involved in the remainder. In their study, a cohort of 252 unrelated patients with hypertrophic cardiomyopathy was screened for variants in several candidate genes involved in calcium regulation, including the *CALR3* gene (MIM 611414). Two heterozygous missense variants c.218G>A p.(Arg73Gln) and c.245A>G p.(Lys82Arg) in *CALR3* were considered of “pathogenic significance”. Both variants were reported to affect conserved amino acids (considering five species) and were not identified in over 200 alleles of healthy individuals. One of the patients also had two potentially disease-causing variants in the *MYBPC3* gene [2].

After the initial publication, *CALR3* has been added to the diagnostic arsenal of many molecular diagnostic laboratories worldwide, including 15 laboratories listed in the Genetic Testing Registry (https://www.ncbi.nlm.nih.gov/gtr) as of 15 April 2018. However, since the initial report, no other studies have confirmed the association between *CALR3* and cardiomyopathy. The protein encoded by the *CALR3* gene, calreticulin-3, belongs to a family of calcium-binding chaperones present in the endoplasmic reticulum. Its exact function remains to be determined. More recent studies have indicated that the calcium-binding capacity of calreticulin-3 is absent or very low [3], and that expression appears to be restricted to the testis [4–7]. Here, we evaluate the genetic and experimental evidence supporting a causal relationship. For this purpose, we assessed the frequency, distribution and potential effect of *CALR3* variants in a Dutch cohort of 6154 cardiomyopathy patients. In addition, we investigated calreticulin-3 protein expression in heart tissue from both patients and controls of different ages. Finally, we discuss our findings in relation to existing literature.

## Materials and methods

### Study population

The study population consisted of 6154 probands with a clinical diagnosis of hypertrophic cardiomyopathy (HCM) [8], dilated cardiomyopathy (DCM) [9], arrhythmogenic right ventricular cardiomyopathy (ARVC) [10], or left ventricular non-compaction (LVNC) [11], referred for genetic testing of cardiomyopathy-related genes in five molecular diagnostic laboratories in the Netherlands between January 2012 and December 2016. Informed consent was obtained from each patient prior to testing. In probands with a rare heterozygous variant in *CALR3*, we evaluated the outcome of cardiac screening and genetic testing in relatives.

### Variant analysis

Genomic DNA was extracted from peripheral blood samples according to standard protocols. Patients were tested for at least 45 cardiomyopathy-related genes that are included in the Dutch core panel: *ABCC9, ACTC1, ACTN2, ANKRD1*, *BAG3, CALR3, CRYAB, CSRP3, DES, DMD, DSC2, DSG2, DSP, EMD, GLA, JPH2, JUP, LAMA4, LAMP2, LDB3, LMNA, MYBPC3, MYH6, MYH7, MYL2, MYL3, MYPN, MYOZ1, MYOZ2, PKP2, PLN, PRKAG2, RBM20, RYR2, SCN5A, SGCD, TAZ, TCAP, TMEM43, TNNC1, TNNI3, TNNT2, TPM1, TTN,* and *VCL*. Genes were analyzed in one of the participating laboratories either by targeted enrichment via hybridization (*n* = 5104) or selective circularization (*n* = 720) techniques, or whole-exome sequencing-based testing of a virtual gene panel (*n* = 330) [12–14]. In this study, we focused on sequence variants in the *CALR3* gene (NG_031959.2, NM_145046.4). The pathogenicity of these variants was assessed using Alamut Visual v2.7.2 software (Interactive Biosoftware, Rouen, France), that integrates data from large-scale population genetic studies, evolutionary conservation of nucleotides and amino acids, in silico missense prediction tools (Align GVGD, SIFT, MutationTaster and PolyPhen-2) and mRNA splicing prediction tools (SpliceSiteFinder-like, MaxEntScan, NNSPLICE, GeneSplicer, and Human Splicing Finder). Potential splice effect was defined as at least 10% difference between reference and mutated scores by at least 3 out of 5 mRNA splicing prediction tools. In addition, the deleteriousness of variants was scored using combined annotation dependent depletion (CADD) [15]. A scaled CADD score of 10, 20, or 30 indicates the top 10%, 1%, and 0.1% most deleterious substitutions in the human genome, respectively. The Mendelian clinically applicable pathogenicity (M-CAP) score was added to improve classification of rare missense variants [16]. A threshold of 0.025 correctly dismisses 60% of variants of unknown significance at 95% sensitivity.

*CALR3* variants with a minor allele frequency (MAF) > 0.1% in the Exome Aggregation Consortium (ExAC) dataset (considering total population and major subpopulations) [17], and synonymous and intronic variants without predicted effect on mRNA splicing were excluded from further analysis. Sanger sequencing was performed to validate the remaining variants and to examine segregation among family members. Additional variants in any of the other cardiomyopathy-related genes were interpreted according to the 2015 American College of Medical Genetics and Genomics/Association of Molecular Pathology (ACMG/AMP) guidelines [18], and classified into five categories: “affects function”, “probably affects function”, “effect unknown”, “probably does not affect function”, and “does not affect function”. The data resulting from this study have been submitted to the corresponding Leiden Open Variant Database at www.LOVD.nl/CALR3 (patient IDs 163857–163904).

### Haplotype analysis

To investigate whether the recurrent c.564del variant originated from a single mutational event, we performed haplotype analysis using eight highly polymorphic microsatellite markers spanning a 1.7 Mb region on chromosome 19p13.11 flanking the *CALR3* gene (D19S588, D19S244, D19S711, D19S917, D19S199, D19S1899, D19S410, and D19S915). Haplotypes were constructed from the genotype data.

### Statistical analysis

Sequence data for 60706 unrelated individuals assembled by ExAC were used as an independent control dataset [19]. Raw data (release 0.3.1) were downloaded and filtered following the same strategy as for the patient cohort. Frequencies were statistically compared using the *χ*^2^ test with Yates’ correction. A *p*-value < 0.05 was considered statistically significant.

### Immunohistochemistry

Immunohistochemical staining was performed on archived myocardial samples from both patients with cardiomyopathy and non-cardiac death. The first group included one individual with c.564del-positive DCM (49 years), and three individuals with ischemic cardiomyopathy (37–62 years). The second group included 12 individuals who died from non-cardiac causes, from four different age groups: fetuses (19–23 weeks of gestation), neonates (6 days–10 weeks), children (7–11 years), and adults (30–57 years). Testicular biopsies were used as positive controls. Samples were handled in accordance with the Code of Conduct for dealing responsibly with human tissue in the context of health research (FEDERA).

Tissue was fixated using formalin 4% (v/v) and embedded in paraffin. The sections (4 µm) were deparaffinized with xylene and rehydrated with ethanol, followed by fully automated antigen retrieval and immunostaining with amplification steps on a Roche Ventana Benchmark Ultra platform (Ventana Medical Systems, Tucson, AZ, USA). Next, sections were incubated during 1 h at room temperature with primary antibodies, including a set of three different polyclonal rabbit antibodies against CALR3: NBP1-33337, NBP2-33390, and NBP2-33524 (Novus Biologicals, Littleton, CO, USA). Negative controls were obtained by omitting the primary antibody. Following visualization with the OptiView detection system (Ventana Medical Systems), slides were examined using a light microscope (Leica Microsystems, Wetzlar, Germany).

## Results

### Genotypic and phenotypic characteristics

**Fig. 1:**
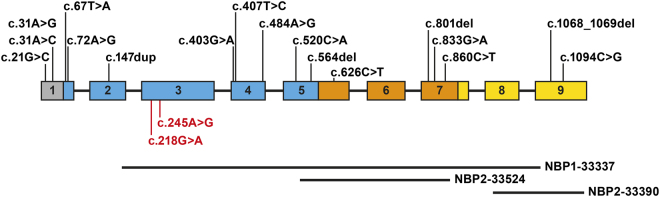
Schematic representation of the *CALR3* gene and corresponding protein domains: signal peptide (gray), N-domain (blue), P-domain (orange) and C-domain (yellow). Boxes represent exons; connecting lines represent intervening introns (unscaled). Rare heterozygous *CALR3* variants identified in this study are indicated in black. Variants reported by Chiu et al. are indicated in red font. The regions recognized by the primary antibodies used in this study are shown below the graph

In our cohort of 6154 patients with cardiomyopathy, we identified 46 unique heterozygous *CALR3* variants. Of these, 17 variants passed our selection criteria, including 12 missense, 1 synonymous, 1 nonsense, and 3 frameshift variants (Table 1; Fig. 1). Eight variants clustered in the N-terminal globular domain (Fig. 1), which is conserved across the calreticulin family. Eight variants were found in more than one family, resulting in a total of 48 probands (Table 1). Phenotypes included asymmetric septal or concentric HCM, left ventricular or biventricular DCM, and LVNC.

**Table 1:** Overview of rare heterozygous *CALR3* variants identified in this study and their associated phenotypes

Nucleotide change	Protein change	# Probands	Phenotype(s)	ExAC MAF	Splice prediction^a^	CADD score	M-CAP score
c.21G>C	p.(Gln7His)	4	HCM, DCM	5/61010	No effect	0.001	0.004
c.31A>G	p.(Ile11Val)	1	HCM	1/62978	No effect	0.001	0.004
c.31A>C	p.(Ile11Leu)	3	HCM, DCM	Absent	No effect	0.001	0.004
c.67T>A	p.(Phe23Ile)	1	HCM	4/52406	No effect	**31**	**0.125**
c.72A>G	p.(Gln24=)	1	HCM	Absent	Effect	1.581	N/A
c.147dup	p.(Arg50*)	2	DCM, LVNC	Absent	No effect	**24.6**	N/A
c.403G>A	p.(Asp135Asn)	7	HCM, DCM, LVNC, mixed	11/108150	No effect	**34**	**0.061**
c.407T>C	p.(Ile136Thr)	2	DCM	Absent	No effect	**27.7**	**0.059**
c.484A>G	p.(Arg162Gly)	2	HCM	Absent	No effect	**23.3**	0.018
c.520C>A	p.(Leu174Ile)	1	LVNC	4/121250	No effect	**26.2**	**0.082**
c.564del	p.(Gln189Serfs*8)	17	HCM, DCM, LVNC, mixed	5/121312	No effect	7.360	N/A
c.626C>T	p.(Thr209Met)	1	HCM	1/121116	No effect	14.36	0.008
c.801del	p.(Glu268Lysfs*13)	1	Mixed	Absent	No effect	4.736	N/A
c.833G>A	p.(Arg278His)	2	HCM, DCM	Absent	No effect	**22.3**	0.017
c.860C>T	p.(Thr287Met)	1	HCM	2/121410	No effect	9.523	0.021
c.1068_1069del	p.(Glu357Glyfs*12)	1	DCM	Absent	No effect	**35**	N/A
c.1094C>G	p.(Ser365Trp)	1	HCM	Absent	No effect	**23.3**	0.015

High pathogenicity scores (CADD ≥ 20 or M-CAP > 0.025) are displayed in bold

Reference sequences: NG_031959.2, NM_145046.4 (*CALR3*)

*CADD* Combined annotation dependent depletion, *DCM* dilated cardiomyopathy, *ExAC* Exome Aggregation Consortium, *HCM* hypertrophic cardiomyopathy, *LVNC* left ventricular non-compaction, *MAF* minor allele frequency, *M-CAP* Mendelian clinically applicable pathogenicity, *N/A* not applicable

^a^Splice effect was defined as at least 10% difference between reference and mutated scores by at least 3 out of 5 mRNA splicing prediction tools (SpliceSiteFinder-like, MaxEntScan, NNSPLICE, GeneSplicer and Human Splicing Finder)

In nine probands (19%), the *CALR3* variant was accompanied by a putative disease-causing variant (“affects function” or “probably affects function”) in another gene of the cardiomyopathy panel (Supplemental Table 1), including a heterozygous Dutch founder variant c.2373dup p.(Trp792Valfs*41) in *MYBPC3* (patient ID 163878, 163893 and 163894), a heterozygous missense variant c.481C>T p.(Pro161Ser) in *MYBPC3* (patient ID 163864), a heterozygous splice variant c.442G>A p.(Gly148Arg) in *MYBCP3* (patient ID 163904), a heterozygous missense variant c.4130C>T p.(Thr1377Met) in *MYH7* (patient ID 163879), a homozygous nonsense variant c.376C>T p.(Gln126*) in *MYL2* (patient ID 163885), a heterozygous nonsense variant in c.814C>T p.(Gln272*) in *TNNT2* (patient ID 163861), and a truncating variant in c.87722_87740del p.(Pro29241Leufs*24) in *TTN* (patient ID 163900). In 17 other probands (35%), we found at least one variant of unknown significance (Supplemental Table 1).

The most common variant, c.564del, was detected in 17 probands from all five laboratories, representing 0.28% of the total cohort and 35% of all detected *CALR3* variants. Two of the probands (patient ID 163881 and 163888), tested in different laboratories, were found to be close relatives. A total of 44 relatives from nine probands had been subsequently tested for the familial variant: 23 relatives had a positive test result, and 21 relatives had a negative test result. Four relatives were obligate heterozygotes. Among the 27 (obligate) heterozygous relatives, 6 (22%) had cardiomyopathy, 17 (63%) had no overt cardiac phenotype, and 4 were not examined (Table 2). Notably, two affected relatives from a large four-generation family tested negative for the c.564del variant. One had severe asymmetric septal hypertrophy (22 mm) in the absence of hypertension. He did not have a variant in any of the other cardiomyopathy genes upon further testing. The second relative had left ventricular hypertrophy with a history or hypertension, and may therefore be considered a phenocopy.

**Table 2:** Genotype–phenotype relationships in relatives from patients with the *CALR3* c.564del variant

	Phenotype positive	Phenotype negative	Phenotype unknown	Total
Genotype positive^a^	6	17	4	27
Genotype negative	2	16	3	21
Total	8	33	7	48

^a^Including four obligate heterozygotes

In five probands with other *CALR3* variants, additional relatives were tested. The *CALR3* variant did not segregate with the phenotype in two of these families. In one family (patient ID 163879), the *CALR3* c.484A>G variant was absent in a sibling whose son had died suddenly of HCM at the age of 23 years. This sibling had, though, inherited the accompanying disease-causing variant in the *MYH7* gene. In a second family (patient ID 163876), the c.407T>C variant was absent in three affected relatives, including a nephew with DCM and out-of-hospital cardiac arrest at the age of 54 years.

### In silico evaluation

Twelve of the *CALR3* variants identified in our patient cohort are predicted to result in an amino acid substitution (Table 1). Six of these variants were absent from ExAC. Four variants had high CADD (≥20) and M-CAP (>0.025) scores, and are therefore more likely to have an effect on the gene or protein function (Table 1).

The c.72A>G variant might affect mRNA splicing by the introduction of a new acceptor splice site. However, the effect could not be tested due to the lack of appropriate mRNA-expressing tissues.

Four variants are predicted to result in a truncated protein. All but one variant (c.564del) were absent from ExAC (Table 1). The c.564del variant predicts a frameshift starting at position Gln189 and resulting in a premature termination codon 7 amino acids downstream: p.(Gln189Serfs*8). This variant was found at low frequency in the ExAC dataset, and was confined to the non-Finnish European population (5/66,682 alleles, MAF = 0.0075%). The variant was absent from the Genome of the Netherlands (GoNL, *n* = 250 parent–child trios) [20], the Rotterdam Study (ERGO, *n* = 2628 elderly individuals from a suburb of the city of Rotterdam) and the Erasmus Rucphen Family study (ERF, *n* = 337 individuals from an isolated population in the South-West of the Netherlands), and present once in our in-house exome sequencing database of healthy parents (1/1642 alleles, MAF = 0.061%).

### Haplotype analysis

Haplotype analysis was performed in 12 of the families with the most frequently observed variant c.564del (Supplemental Table 2). A shared haplotype for at least 4 of the 8 markers, located in a 1.2 Mb region surrounding the *CALR3* gene, was observed in 6 families, suggesting that the variant has arisen through a single mutational event in a common ancestor. Therefore, it seems reasonable to exclude this variant from the burden test (see below).

### Burden test

The observed number of missense and truncating *CALR3* variants in ExAC is approximately equal to the expected number (*Z*-score −0.10 and pLI 0.00, respectively), indicating that these variants seem to be tolerated [19]. Following the same filtering steps used in our patient cohort, the ExAC dataset contained 139 variants with potential functional impact, including 118 missense, 2 synonymous, 8 nonsense, 2 start loss, 5 splice site, and 4 frameshift variants. Approximately half of these variants (64/139 = 46%) were located in the conserved N-domain, as was observed in our patient cohort. The 139 variants were present in 298/121,412 alleles, corresponding to 0.49% of the control cohort. In comparison, we found 17 variants in 48/12,308 alleles, corresponding to 0.78% of the patient cohort (*p* = 0.0036). After excluding the c.564del variant, which should probably be considered as a single mutational event (see above), no statistically significant difference was observed (*p* = 0.89). Likewise, no difference was observed when only missense variants were taken into account (*p* = 0.93).

### Protein expression analysis

**Fig. 2:**
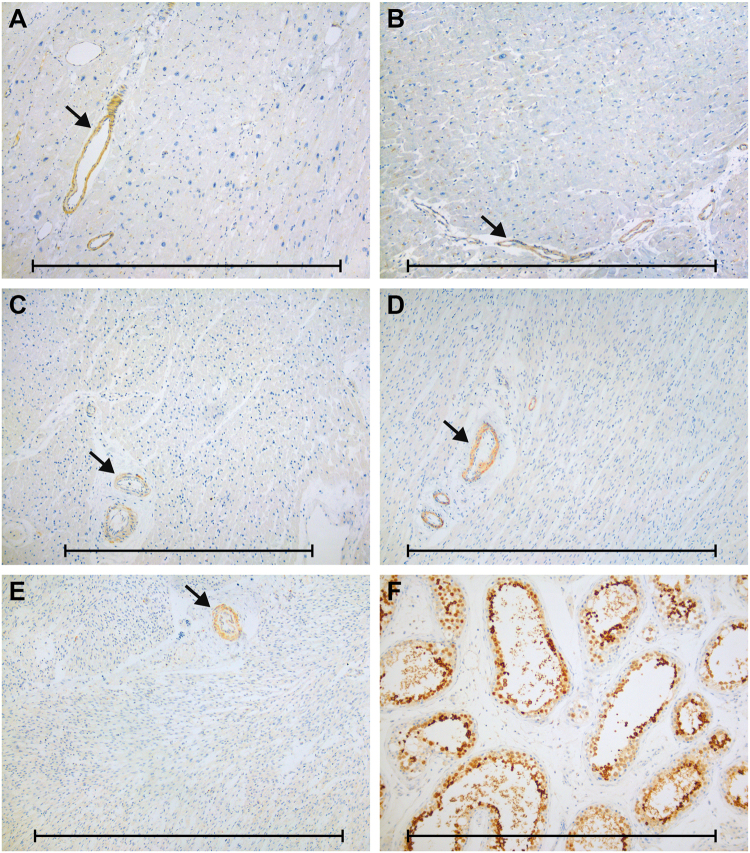
Examples of immunohistochemical staining for CALR3 (antibody NBP2-33390) in normal and affected tissues. No expression in myocardial tissue from (**a**) patient with c.564del-positive DCM, **b** patient with ischemic cardiomyopathy, and **c** child, **d** newborn, and **e** fetus with structurally normal hearts. Instead, we observed positive staining of arteriolar smooth muscle cells in all samples (arrows). **f** High expression of CALR3 in testicular germ cells as positive control tissue. Scale bars: 1 mm

Previous studies, including the comprehensive collection of data from the Genotype-Tissue Expression (GTEx) project and the Human Protein Atlas (HPA), have demonstrated testis-specific expression of CALR3 mRNA and protein in adult human tissues [4–7]. To verify that CALR3 is not expressed in the heart, in particular at earlier time points, we performed immunohistochemical staining in a series of myocardial tissues (from fetuses to adults) using a set of three different antibodies against the calreticulin-3 protein. Testicular samples were used as positive controls. Calreticulin-3 expression was virtually absent in myocardial tissues from each group (Fig. 2a–e). We only observed some antibody-dependent staining of vascular smooth muscle cells of arterioles (NBP2-33390), stromal cells (NBP2-33390), and mast cells (NBP1-33337). All three antibodies showed high expression of the CALR3 protein in testicular germ cells (Fig. 2f), as described previously [6, 7].

## Discussion

We investigated the frequency and characteristics of *CALR3* variants in a large Dutch cardiomyopathy cohort. Based on several observations in this study, we question the impact of *CALR3* genetic variation on cardiomyopathy. (i) In approximately one-fifth of the probands, the *CALR3* variant was accompanied by a disease-causing variant in another, well-known cardiomyopathy gene. Another 35% of probands had at least one variant of unknown significance in one of the other panel genes. (ii) In 3 out of 14 families investigated in further detail, the *CALR3* variant did not segregate with the disease. (iii) After exclusion of a Dutch founder variant, no statically significant difference was found in allele frequencies between our patient cohort and the ExAC dataset (*p* = 0.89). (iv) We could not detect CALR3 protein expression in myocardial tissue at various ages. This observation is in line with previous studies, that reported testis-specific expression [4–7].

Calreticulin-3 (also known as calsperin or calreticulin-2) belongs to a family of calcium-binding chaperones localized in the lumen of the endoplasmic reticulum. *CALR3* is mainly expressed in the reproductive system [6, 7, 21]. Its exact role in both health and disease is still largely unknown. Calreticulin-3 contains three functional domains: a N-terminal globular domain, a proline-rich P-domain, and a C-terminal acidic domain. The N-domain has a conserved amino acid sequence with binding sites for zinc ions and glycans. In contrast to other members of the calreticulin family, the calcium-binding capacity of the P-domain and the C-domain seems absent or low [3]. Its paralog calreticulin (*CALR*), on the other hand, encodes a ubiquitously expressed protein that has been implicated in a variety of cellular processes, including calcium homeostasis, protein folding, and cell adhesion [22].

Chiu et al. were the first to investigate the role of *CALR3* in human, assuming that variants in this gene may lead to calcium dysregulation with subsequent HCM. However, their assumption is based on studies concerning the multifunctional and ubiquitously expressed paralog *CALR*. Two heterozygous missense variants in *CALR3* (c.218G>A p.(Arg73Gln) and c.245A>G p.(Lys82Arg)) were identified in two unrelated patients with HCM [2]. The pathogenicity of both variants can be questioned based on current data resources. The c.218G>A variant affects a conserved nucleotide and amino acid (considering 17 species). The physicochemical difference between arginine and glutamine is small (Grantham distance 34). PolyPhen-2 is the only algorithm predicting a damaging effect. The variant is observed at low frequency in ExAC (4/121412 alleles, MAF = 0.0033%). The proband had two additional variants in *MYBPC3*, c.2234A>G p.(Asp745Gly) and c.2618C>A p.(Pro873His), which are present in the Human Gene Mutation Database and more likely to account for the HCM. The other *CALR3* variant c.245A>G also affects a conserved nucleotide and amino acid. The physicochemical difference between lysine and arginine is small (Grantham distance 26). The variant is observed at relatively high frequency in ExAC (69/121412 alleles, MAF = 0.057%), and is therefore considered unlikely to be the cause of the disease [23, 24].

The *CALR3* gene has been studied in several animal models. Male *Calr3* knockout mice were infertile due to defective sperm migration and binding to the zona pellucida; no other gross abnormalities were observed, specifically no cardiac dysfunction [21]. Female *Calr3* knockout mice were completely normal. Zebrafish have two orthologs of *CALR3*: *calr3a* (NM_131047.2) and *calr3b* (NM_201465.3). Knockdown of *calr3a* using antisense morpholino oligonucleotides causes a reduction of posterior lateral line neuromasts; superficial sensory organs that enable the fish to detect changes in water flow. Cardiac abnormalities were not reported [25]. No phenotypic data are available on *calr3b* mutants. *Calr* knockout mice, on the other hand, display impaired cardiac development and function, resulting in early embryonic lethality [26]. Transgenic mice overexpressing calreticulin show progressive conduction abnormalities, leading to complete heart block and early postnatal death [27]. These data suggest that *CALR*, rather than *CALR3*, is a good candidate gene for cardiac disease in humans.

### Study limitations

Immunohistochemical staining showed absence of calreticulin-3 expression in second-trimester fetal myocardium. However, we cannot exclude the possibility that calreticulin-3 is expressed at earlier stages of cardiac development. In addition, although our data do not support a role of *CALR3* in cardiomyopathy as single-gene disorder, a more complex pattern of inheritance cannot be ruled out.

## Conclusions

Based on our findings, we highly question the implication of *CALR3* in cardiomyopathy. Our data suggest that *CALR3* variants are not monogenic causes of cardiomyopathy, if cardiovascular disease-related at all.

## Electronic supplementary material

Supplemental Material
