# Supplementary material for: Lack of evidence for a causal role of CALR3 in monogenic cardiomyopathy
Source: Eur J Hum Genet. 2018 Jul 9;26(11):1603–10. doi: 10.1038/s41431-018-0208-1 (PMC6189092; doi:10.1038/s41431-018-0208-1)
Supplement: Supplementary file 1 — Supplemental Material [file 41431_2018_208_MOESM1_ESM.docx]

**SUPPLEMENTARY DATA**

**Lack of evidence for a causal role of *CALR3* in monogenic cardiomyopathy**

Judith M.A. Verhagen, Job H. Veldman, Paul A. van der Zwaag, Jan H. von der Thüsen, Erwin Brosens, Imke Christiaans, Dennis Dooijes, Apollonia T.J.M. Helderman-van den Enden, Ronald H. Lekanne Deprez, Michelle Michels, Anneke M. van Mil, Rogier A. Oldenburg, Jasper J. van der Smagt, Arthur van den Wijngaard, Marja W. Wessels, Robert M.W. Hofstra, Marjon A. van Slegtenhorst, Jan D.H. Jongbloed and Ingrid M.B.H. van de Laar

**Supplementary Table 1.** Classification of additional variants identified in this study according to the 2015 ACMG/AMP criteria

| **Gene** | **Nucleotide change** | **Protein change** | **Zygosity** | **Effect** | **Evidence** | **Reference** |
| --- | --- | --- | --- | --- | --- | --- |
| *ABCC9* | c.3640G>A | p.(Ala1214Thr) | Heterozygous | Effect unknown | PM1 PM2 PP3 BP5 |  |
| *ACTN2* | c.1342G>C | p.(Glu448Gln) | Heterozygous | Effect unknown | PM2 PP3 |  |
| *CTNNA3* | c.457G>C | p.(Ala153Pro) | Heterozygous | Effect unknown | BP4 |  |
|  | c.1133G>A | p.(Arg378His) | Heterozygous | Probably does not affect function | PP3 BS1 BP6 |  |
|  | c.2378G>A | p.(Gly793Glu) | Heterozygous | Probably does not affect function | BS1 BP5 |  |
| *DES* | c.934G>A | p.(Asp312Asn) | Heterozygous | Effect unknown | PM1 PP3 BS1 | Taylor et al., 2007 |
| *DSC2* | c.942+3A>G | p.? | Heterozygous | Effect unknown | PM2 BP5 | Cox et al., 2011 |
| *DSP* | c.5449G>A | p.(Val1817Met) | Heterozygous | Effect unknown | PM2 BP4 |  |
| *HCN4* | c.1996C>T | p.(Arg666Trp) | Heterozygous | Effect unknown | PM1 PM2 PP3 |  |
| *LAMA4* | c.578C>T | p.(Ser193Leu) | Heterozygous | Effect unknown | PM1 |  |
|  | c.4636G>A | p.(Glu1546Lys) | Heterozygous | Effect unknown | PM1 PM2 |  |
| *LDB3* | c.608C>T | p.(Ser203Leu) | Heterozygous | Effect unknown | PM2 PP3 |  |
| *MYBPC3* | c.442G>A | p.(Gly148Arg) | Heterozygous | Affects function | PVS1 PP1(S) PS3 | Zimmerman et al., 2010 |
|  | c.481C>T | p.(Pro161Ser) | Heterozygous | Probably affects function | PP1(S) PM2 PP3 PP5 | Alders et al., 2003 |
|  | c.2373dup | p.(Trp792Valfs*41) | Heterozygous | Affects function | PVS1 PS4 PP1(S) PP5 | Alders et al., 2003 |
|  | c.3323A>C | p.(Lys1108Thr) | Heterozygous | Effect unknown | PM1 PM2 PP3 |  |
| *MYH6* | c.3932C>T | p.(Thr1311Ile) | Heterozygous | Probably does not affect function | PM1 PP3 BS1 BP5 |  |
| *MYH7* | c.4130C>T | p.(Thr1377Met) | Heterozygous | Probably affects function | PS4 PM2 PP3 | Kelly et al., 2018 |
|  | c.5704G>C | p.(Glu1902Gln) | Heterozygous | Probably does not affect function | BS1 BP5 | Chiou et al., 2015 |
| *MYL2* | c.376C>T | p.(Gln126*) | Homozygous | Affects function | PVS1 PS3 PM2 PP3 |  |
| *MYPN* | c.3335C>T | p.(Pro1112Leu) | Heterozygous | Effect unknown | PM1 PP3 BS1 | Duboscq-Bidot et al., 2008 |
| *PKP2* | c.101C>A | p.(Ser34Tyr) | Heterozygous | Effect unknown | PM2 BP1 |  |
| *RBM20* | c.773C>T | p.(Ser258Leu) | Heterozygous | Effect unknown | PM2 |  |
| *SCN5A* | c.3578G>A | p.(Arg1193Gln) | Heterozygous | Probably does not affect function | BS1 BP4 BP5 | Vatta et al., 2002 |
| *TAZ* | c.774G>A | p.(Ser258Ser) | Hemizygous | Probably does not affect function | PM2 BS2 BP7 |  |
| *TNNT2* | c.410A>T | p.(Gln137Leu) | Heterozygous | Effect unknown | PM2 PP3 |  |
|  | c.431G>A | p.(Arg144Gln) | Heterozygous | Effect unknown | PM2 |  |
|  | c.814C>T | p.(Gln272*) | Heterozygous | Probably affects function | PVS1 PM2 |  |
| *TTN* | c.24083G>C | p.(Gly8028Ala) | Heterozygous | Effect unknown | PM2 PP3 BP1 |  |
|  | c.29503T>C | p.(Tyr9835His) | Heterozygous | Effect unknown | PM2 PP3 BP1 |  |
|  | c.31763-1G>A | p.? | Heterozygous | Effect unknown | PVS1 BS1 | Roberts et al., 2015 |
|  | c.38378A>G | p.(Lys12793Arg) | Heterozygous | Probably does not affect function | BS1 BP1 BP5 |  |
|  | c.52139A>T | p.(Asp17380Val) | Heterozygous | Effect unknown | PP3 BP1 |  |
|  | c.82610G>A | p.(Gly27537Asp) | Heterozygous | Effect unknown | PM2 BP1 |  |
|  | c.87722_87740del | p.(Pro29241Leufs*24) | Heterozygous | Probably affects function | PVS1 PM2 | Jansweijer et al., 2017 |
|  | c.88496T>G | p.(Leu29499Arg) | Heterozygous | Effect unknown | PP3 BP1 |  |
|  | c.93524G>A | p.(Arg31175His) | Heterozygous | Probably does not affect function | BS1 BP1 |  |
|  | c.96182T>C | p.(Ile32061Thr) | Heterozygous | Effect unknown | PP3 BP1 |  |
|  | c.102271C>T | p.(Arg34091Trp) | Heterozygous | Probably does not affect function | BS1 BP1 BP5 | Lange et al., 2005 |
|  | c.107105C>T | p.(Pro35702Leu) | Heterozygous | Effect unknown | BP1 |  |

Reference sequences: NM_020297.2 (*ABCC9*), NM_001103.3 (*ACTN2*), NM_013266.3 (*CTNNA3*), NM_001927.3 (*DES*), NM_004949.4 (*DSC2*), NM_004415.3 (*DSP*), NM_005477.2 (*HCN4*), NM_001105206.2 (*LAMA4*), NM_007078.2 (*LDB3*), NM_000256.3 (*MYBPC3*), NM_002471.3 (*MYH6*), NM_000257.3 (*MYH7*), NM_000432.3 (*MYL2*), NM_032578.3 (*MYPN*), NM_004572.3 (*PKP2*), NM_001134363.2 (*RBM20*), NM_198056.2 (*SCN5A*), NM_000116.3 (*TAZ*), NM_001001430.2 (*TNNT2*), NG_011618.3, NM_001267550.2 (*TTN*). Note: PM2 was applied when a variant was absent or extremely rare (<0.004%) in large population cohorts, as proposed by ClinGen’s Inherited Cardiomyopathy Expert Panel (Kelly et al, 2018: PMID 29300372) .

**Supplemental Table 2.** Haplotype associated with the *CALR3* c.564del variant in the index patients

| **Marker** | **Position** | **A-III:1** | | **B-III:2** | | **C-II:1** | | **D-II:1** | | **E-III:1** | | **F-II:1** | | **G-II:1** | | **H-II:1** | | **I-II:2** | | **J-II:1** | | **K-II:1** | | **L-II:1** | |
| --- | --- | --- | --- | --- | --- | --- | --- | --- | --- | --- | --- | --- | --- | --- | --- | --- | --- | --- | --- | --- | --- | --- | --- | --- | --- |
| D19S588 | 15628156 | 169 | 169 | 169 | 149 | 149 | 173 | 169 | 169 | 169 | 165 | 149 | 149 | 169 | 149 | 169 | 153 | 169 | 173 | 169 | 149 | 169 | 153 | 169 | 153 |
| D19S244 | 15667226 | 100 | 108 | 100 | 104 | 108 | 140 | 112 | 124 | 100 | 136 | 108 | 104 | 100 | 112 | 112 | 128 | 100 | 100 | 100 | 108 | 112 | 144 | 108 | 136 |
| D19S711 | 15992789 | 167 | 155 | 167 | 170 | 167 | 164 | 161 | 161 | 167 | 161 | 166 | 164 | 167 | 164 | 158 | 170 | 167 | 161 | 167 | 161 | 167 | 167 | 161 | 158 |
| D19S917 | 16261017 | 225 | 225 | 225 | 221 | 225 | 235 | 225 | 227 | 225 | 228 | 225 | 228 | 225 | 228 | 227 | 227 | 225 | 227 | 225 | 225 | 225 | 225 | 225 | 225 |
| CALR3 | c.564del | + | - | + | - | + | - | + | - | + | - | + | - | + | - | + | - | + | - | + | - | + | - | + | - |
| D19S199 | 16868262 | 168 | 162 | 168 | 162 | 168 | 154 | 162 | 146 | 166 | 152 | 164 | 164 | 168 | 162 | 162 | 148 | 166 | 148 | 166 | 146 | 164 | 146 | 166 | 162 |
| D19S899 | 17134083 | 115 | 113 | 107 | 115 | 113 | 105 | 105 | 111 | 115 | 113 | 103 | 111 | 115 | 103 | 113 | 113 | 115 | 121 | 113 | 111 | 113 | 121 | 105 | 111 |
| D19S410 | 17297335 | 162 | 168 | 166 | 166 | 158 | 160 | 164 | 164 | 162 | 167 | 164 | 167 | 162 | 153 | 166 | 166 | 162 | 152 | 162 | 164 | 166 | 152 | 170 | 170 |
| D19S915 | 17817874 | 108 | 92 | 108 | 102 | 108 | 108 | 110 | 102 | 90 | 112 | 102 | 102 | 102 | 108 | 90 | 106 | 90 | 102 | 90 | 102 | 110 | 112 | 90 | 102 |

Values represent the length of amplified fragments. The shared haplotype surrounding the *CALR3* c.564del variant is marked in grey. In families A, B, C, D and E more than one family member was genotyped. For clarity, only the index patients are displayed here.
